# Cervicomediastinal Giant Cystic Hygroma: A Case Report

**DOI:** 10.4021/jocmr1089w

**Published:** 2013-01-11

**Authors:** Omer Karakas, Ekrem Karakas, Fatima Nurefsan Boyaci, Murat Yildizhan, Songul Demir, Mehmet Akif Saglam, Nesat Cullu

**Affiliations:** a Department of Radiology, Harran University, Faculty of Medicine, Turkey; b Department of Radiology, Mugla Sitki Kocman University, Faculty of Medicine, Turkey

**Keywords:** Cystic hygroma, Mediastinum, Neck, MRI

## Abstract

Cystic hygroma is a rare congenital malformation of the lymphatic system. It is most frequently detected in the head and neck region. Ultrasonography is performed as the first step for radiological diagnosis of these lesions. Magnetic resonance imaging provides important information for diagnosis and to guide treatment. In this paper, a case is reported of cervicomediastinal large cystic hygroma in a male infant.

## Introduction

Cystic hygroma is a rarely encountered congenital malformation of the lymphatic system, which is most often observed in the head and neck region. Ultrasonography (US) is used as the first step in radiological diagnosis of these lesions. However, magnetic resonance imaging (MRI) is being more frequently used to evaluate the lesion structure, dimensions and extent and to determine the treatment plan and appropriate treatment approach [1-4]. The aim of this case report was to present the MRI findings of an infant with cervicomediastinal cystic hygroma, together with the relevant literature.

## Case Report

A 2-year-old male patient presented at the clinic with complaints of a swelling on the left side of the neck, which had been present since birth and seemed to have increased in size over the previous 2 - 3 months. Physical examination determined a mass lesion in the left cervical region reaching approximately 6 cm in size, slightly mobile and pressurized, with no changes in skin color or temperature, leading to posture impairment of the neck. In the laboratory findings, in the acute phase reactants there was a slight increase in lymphocytes and a mild level of iron deficiency anemia. Other routine laboratory results were normal. B-mode USG examination was directed to the cervical region. A complex cystic lesion was observed to be located in the left anterior cervical region, extending intrathoracically.

**Figure 1:**
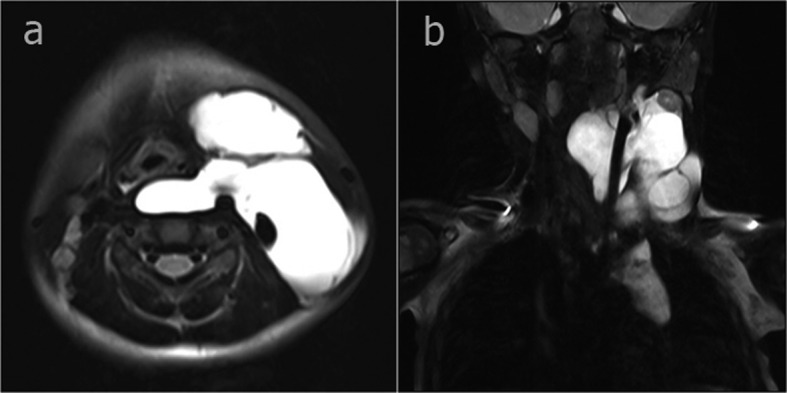
In the axial fat-suppressed T2-weighted image (a) a hyperintense cystic mass wrapped around the internal jugular vein, extending to the retropharyngeal area in the left cervical region (b) a hyperintense cystic mass wrapped around the internal jugular vein, extending to the mediastinum from the left cervical region.

**Figure 2:**
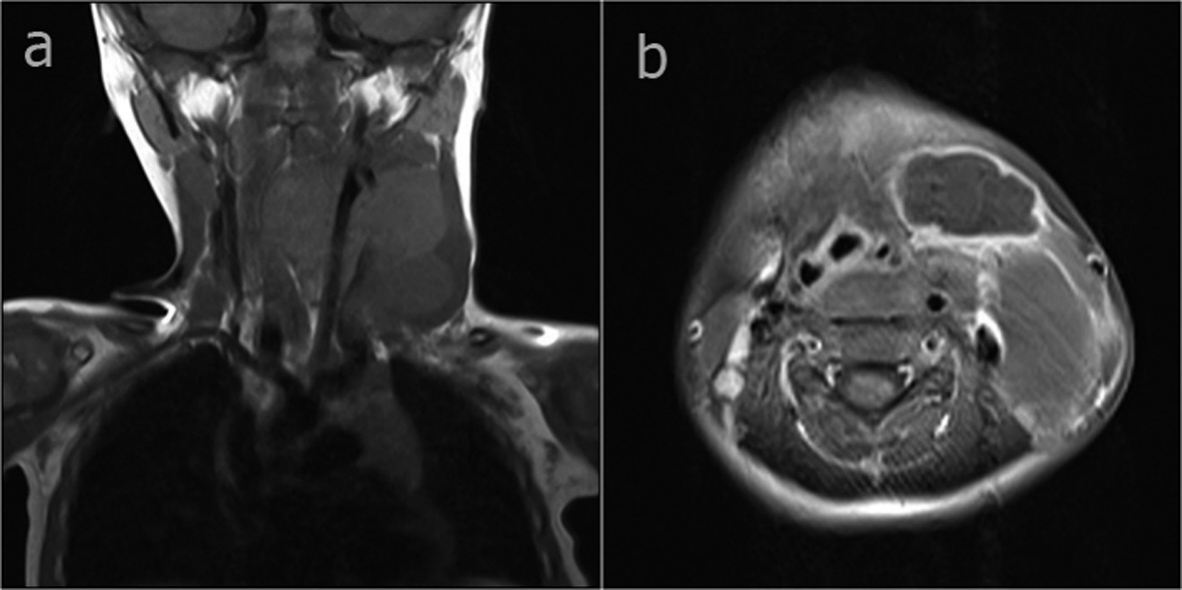
In the coronal T1-weighted image (a) a cystic mass wrapped around the internal jugular vein on intermittent signal dependent on the haemorrhagic and/or proteinous content, extending to the mediastinum from the left cervical region. In the axial fat-suppressed T1-weighted image (b) cystic mass showing contrast observed in the wall of the left cervical region.

MRI examination was made to evaluate the extent of the lesion and details of the internal structure. From the MRI examination, the lesion was determined to be located on the left half of the neck in the infra-suprahyoid region, extending into the retropharyngeal cavity, to the posterior cervical triangle and the intrathoracic left superior mediastinum. The internal jugular vein was completely surrounded by the lesion. The multilocular, septated lesion with lobular contours and thick walls was separated from the surrounding tissue with clear borders. In T1-weighted images, the lesion was seen on intermittent signals depending on hemorrhagic and/or proteinous content and on T2-weighted fat suppressed images there was evident hyperintensity. On contrast T1-weighted fat suppressed images there was only contrast of the thick septa and wall (Fig. 1a, 1b, 2a, 2b). Although neighboring anatomic structures were compressed due to the mass effect of the lesion, there was no significant compression effect. These findings together with the cervicomediastinal location led to an initial diagnosis of cystic hygroma being considered. The cytology result of aspiration from the lesion was consistent with cystic hygroma. As the lesion was widespread, wrapped around vascular structures and of a macrocystic structure, the decision was made for percutaneous treatment. The lesion contents were aspirated percutaneously. In the same session an injection of sclerosing substance (bleomycin) was administered. At the 3-month follow-up examination, the lesion was determined to have nearly completely reduced.

## Discussion

Cystic hygroma (lymphangioma) is a rarely seen congenital malformation of the lymphatic system, which most often involves the head and neck region [1-4]. Half of cases are determined at birth and most are diagnosed under the age of two years [5]. The case presented here had had a mass lesion since birth and a diagnosis of cystic hygroma was made at the age of two years.

In the diagnosis of cystic hygroma, US, CT and MRI can be used. Various thicknesses of septa are seen as anechoic, multilocular cystic masses on US. However, US is not sufficient to extend to the retropharyngeal, axillary and mediastinal view [6]. On CT images, cystic masses of low density depending on the fluid content are seen with regular and lobular contours. However, the CT density of a cystic hygroma may be high because of protein, hemorrhaging, fat and other solid contents [7, 8]. MRI is of great benefit in the correct evaluation of the lesion dimensions, internal structure and extent of cystic hygroma located in the cervical region and in the selection of the correct treatment. Surrounding muscle tissue is observed with matched signals on T1-weighted MR images and on T2-weighted images with high signals according to the fat tissue. However, MR signals of cystic hygroma may be heterogeneous because of protein, hemorrhage, fat and other solid contents [7, 8]. Compared to CT, MRI shows the septa and vascular type structures of cystic hygroma more clearly [8]. In the case presented here, there was an image of a cystic hygroma located in the left anterior cervical region extending intrathoracically as a complex cystic mass. The intermittent signal intensity on the T1-weighted images was dependent on the proteinous and hemorrhagic content. On the contrast MR images there was mild contrast of the wall and septa.

Small lymphangioma which do not create functional impairment or aesthetic problems do not require interventional treatment. However, treatment is unavoidable for lesions located in the cervicomediastinal region, particularly when respiration and feeding are inhibited. Most lesion treatment is based on surgical removal [5, 9]. Another treatment modality is percutaneous aspiration of the lesion followed by an injection of a sclerosing agent (sclerotherapy) [10-14]. In the case presented here, as the lesion was of macrocystic structure and located in both the cervical and thoracic region, percutaneous aspiration was applied with ultrasonography and sclerotherapy with bleomycin. The greater part of the lesion was emptied by aspiration. Treatment was completed by injection of a sclerosing agent throughout the whole cyst.

It can be concluded that in cystic hygroma located in the cervical region, MRI examination is of great benefit for correct evaluation of the lesion extent, dimensions and internal structure and thus selection of the correct treatment.
